# Can Simulated Microgravity and Darkness Conditions Influence the Phytochemical Content and Bioactivity of the Sprouts?—A Preliminary Study on Selected Fabaceae Species

**DOI:** 10.3390/plants13111515

**Published:** 2024-05-30

**Authors:** Marta Grudzińska, Agnieszka Galanty, Ewelina Prochownik, Agata Kołodziejczyk, Paweł Paśko

**Affiliations:** 1 Doctoral School of Medical and Health Sciences, Jagiellonian University Medical College, 16 Łazarza St., 31-530 Cracow, Poland; marta.grudzinska@doctoral.uj.edu.pl; 2 Department of Food Chemistry and Nutrition, Jagiellonian University Medical College, Medyczna 9, 30-688 Cracow, Poland; ewelina.gajdzik@uj.edu.pl; 3 Department of Pharmacognosy, Jagiellonian University Medical College, Medyczna 9, 30-688 Cracow, Poland; agnieszka.galanty@uj.edu.pl; 4 Space Technology Centre, AGH University of Technology, 36 Czarnowiejska St., 30-054 Cracow, Poland; akolodziejczyk@agh.edu.pl

**Keywords:** *Fabaceae*, sprouts, simulated microgravity, cytotoxic, antioxidant, isoflavones, phenolics

## Abstract

Sprouts’ consumption has become popular due to their wide availability, easy cultivation process, and proven biological activity. Moreover, stress factors, such as limited access to light or disturbed gravity during growth, may contribute to the increased activity and the synthesis of bioactive compounds. In this study, for the first time, the examination of the impact of darkness and simulated microgravity conditions on the white clover sprouts from the *Fabaceae* family was conducted. Among several species, used in the preliminary attempts, only white clover was satisfactory sprouting in the disturbed gravity conditions, and thus was chosen for further examination. A random positioning machine setup was used during the cultivation process to simulate microgravity conditions. Additionally, the sprouts were cultivated in total darkness. Simulated microgravity and/or darkness during the first few days of the sprouts’ growth caused biomass reduction, the increased synthesis of bioactive compounds (isoflavones and phenolics), and changes in the level of abscisic acid and phenylalanine ammonia-lyase. Moreover, it increased the antioxidant properties of the sprouts, while the enhancement of their cytotoxic impact was observed only for androgen-dependent prostate cancer LNCaP cells. To conclude, the presented results are promising in searching for novel functional food candidates and further studies are necessary, directed at other plant families.

## 1. Introduction

An increasing trend in the daily consumption and variety of different types of sprouts available in grocery stores is accompanied by a growing number of publications on the biological properties, such as antioxidant, anti-inflammatory, and cytotoxic effects, of these new elements of the human diet. Sprouts, being a part of the plant with unique concentrations of active compounds, may have significant chemopreventive potential, as confirmed in the recent systematic review published by our research group [1].

It is worth adding that sprouts are a good source of bioactive phytochemicals from different groups, and their cultivation process is quick and simple; they can also be easily purchased in stores around the world or grown at home within a few days. It was decided to focus our study on the representatives of the *Fabaceae* family, famous for their beneficial health effects, with isoflavones as the most important bioactive compound. This particular chemical group is characterized by estrogenic properties, used in the support of some hormonal disorders like menopause [2].

There are a lot of different methods to stimulate the synthesis of active compounds during sprouts’ growth, with special emphasis on LED lights [3,4], salinity [5], or disturbed gravity. The use of altered gravity and its impact on growth, cellular changes, and plant orientation was extensively studied [6,7] but in the case of sprouts, this method was implemented only rarely. According to one of the recent studies [8], microgravity conditions, a phenomenon described as a state of space with a decreased gravitational acceleration, can enhance the growth of mung bean seedlings, a plant from the *Fabaceae* family, like clover. Moreover, microgravity caused an increase in the antioxidant activity and the content of phenolic compounds in 4-days-old seedlings of mung bean [9]. In turn, Wakabayashi et al. (2005) observed an increase in the concentration of phenolic acids in 3-days-old wheat seedlings growing in hypergravity conditions [10]. *Pinus pinea* seedlings showed higher contents of enzymes involved in seed germination in microgravity conditions, but lower contents in hypergravity conditions, and the results indicate that microgravity can stimulate plant growth [11]. These results inspired us to explore the issue, especially since, apart from the mentioned work by Nakajima et al. (2019), we did not find any results regarding the impact of disturbed gravity on the biological activity of sprouts [9].

The main goal of the study was to investigate whether disturbed gravity and/or total darkness are stress factors for growing sprouts and can influence the synthesis of bioactive ingredients, which in turn contributes to the increase in their biological activity, such as cytotoxic or antioxidant properties. Additionally, we wanted to evaluate if the length of cultivation (5 to 7 days) may result in the changes in phytochemical content of the sprouts, and thus influence the biological activity. To do this, the seeds of several species from the *Fabaceae* family (e.g., clovers, soy, lentil, and beans) were cultivated in the conditions of simulated microgravity obtained using a random positioning machine (RPM) and the control conditions of normal gravity. It was decided to harvest the sprouts in standard light conditions and total darkness. We measured the biomass changes; the amount of abscisic acid (ABA), which is a plant stress-related hormone [12]; and the activity of phenylalanine ammonia-lyase (PAL), involved in isoflavone synthesis [13]. Quantitative analysis was directed to isoflavones and phenolic compounds, followed by the determination of antioxidant (FRAP and DPPH) properties and cytotoxic activity against hormone-dependent and independent prostate (LNCaP, PC3, and DU145) and breast (MCF7 and MDA-MB-231) cancer cells. Such choice of the cell lines was justified by isoflavone presence in white clover sprouts, which may reveal a chemopreventive effect towards the hormone-dependent cancers of the breast and prostate. As a final step, the safety of the obtained sprout extracts to non-cancerous prostate (PNT2) and breast (MCF10A) cells was also determined.

Thanks to these preliminary studies, it will be possible to assess the impact of disturbed gravity and darkness on the biological activity of the selected sprouts from the *Fabaceae* family. In addition, a pre-selection of plant species will be made, which, grown under microgravity conditions, could be new candidates for functional food.

## 2. Results and Discussion

Our original research plan included the use of a number of species from the *Fabaceae* family to be grown in simulated microgravity conditions, namely white, Persian, red, and crimson clovers; vetch; alfalfa; soy; lentil; fenugreek; lupine; green peas; chickpea; mung; and adzuki beans. However, except for the white clover, the seeds of the examined species did not show the desired germination parameters during the experiment in simulated microgravity conditions. Most of them did not start sprouting even after a few days of cultivation, and larger seeds, such as chickpeas or adzuki beans, began to release starch and ferment as a result of the seeds hitting the walls of the containers during the rotations performed by RPM. Thus, further research was performed on the white clover sprouts.

### 2.1. Growth Parameters, Biomass, and Germination Rate

**Figure 1:**
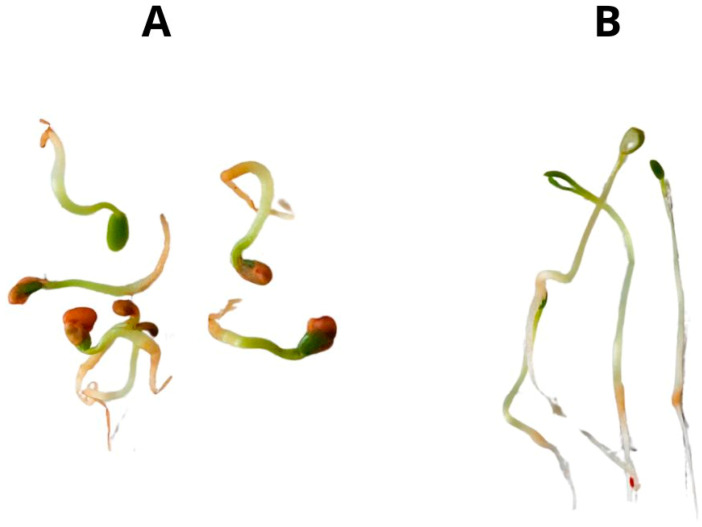
White clover sprouts on the 6th day of germination in simulated microgravity ((**A**), sample 6LM) and normal gravity conditions ((**B**), sample 6L). Abbreviations of the sprouts’ samples: 6LM—6-days-old sprouts grown in standard light and simulated microgravity conditions; 6L—6-days-old sprouts grown in standard light conditions.

In the first step of the experiment, we observed that the growth of the sprouts in the simulated microgravity conditions was clearly reduced, when compared to the control sprouts, growing in normal gravity conditions. The simulated microgravity caused the roots and stems of the sprouts to be much shorter and curled (Figure 1A), compared to control conditions in which the sprouts were much longer and with straight stems (Figure 1B).

Particular growth parameters such as seedling, stem, and root length are presented in Table 1. The highest seedling lengths were in samples 6L, 7L, 6D, and 7D, which were cultivated in normal gravity in both standard light and darkness. The same samples but cultivated in microgravity conditions (6LM, 7LM, 6DM, and 7DM) were characterized by a statistically significant decrease in seedling length. The highest stem lengths were observed in 7L, 6D, and 7D samples and they were statistically significant. As far as root length is concerned, the microgravity and/or darkness conditions did not affect this parameter in the tested sprouts. An obvious observation is also the increasing length of seedlings with the elongation of the cultivation time.

Based on these results, it can be concluded that microgravity and darkness conditions during cultivation slow the growth of the sprouts, which can also be confirmed by the results of biomass accumulation presented below.

**Figure 2:**
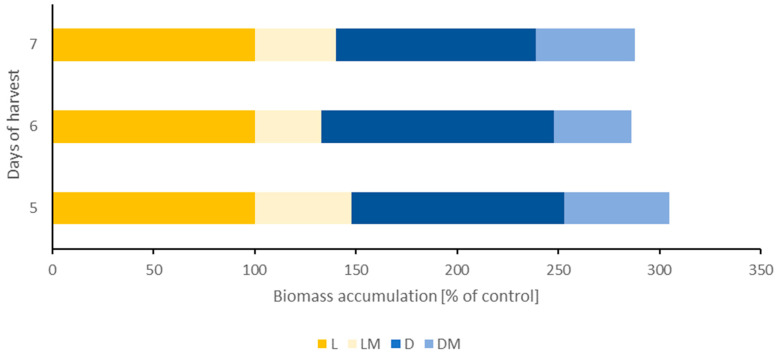
Changes in the biomass of the white clover sprouts presented as % of control (weight of control sprouts 5L, 6L, and 7L). Abbreviations of the sprouts’ samples: L—the sprouts grown in standard light conditions; D—the sprouts grown in darkness; LM—the sprouts grown in standard light and simulated microgravity conditions; DM—the sprouts grown in darkness and simulated microgravity conditions.

The differences in biomass between the tested sprouts are presented in Figure 2. The results clearly indicate that simulated microgravity conditions during sprouting significantly decreased the biomass of the white clover sprouts, observed both in standard light and darkness conditions, up to even 51.5% in sample 5DM. Our results are consistent with Ivanova et al. (1993), who concluded that plants’ biomass production and plants’ growth in the “SVET” greenhouse during space flight was decreased compared to the control group [14]. On the other hand, Nakajima et al. (2021) observed an increase in the weight and length of the clinostat (a device that rotates in order to induce disturbed gravity) grown seedlings of mung beans in comparison to the control ones [8]. Similarly, Kruse et al. (2020) noted that microgravity enhanced the biomass production and growth of *Arabidopsis thaliana* seedlings [15]. Additionally, lack of sunlight (darkness conditions) caused the yellowing of the leaves, probably due to the decreased chlorophyll synthesis, and an increase in biomass production only in 6-days-old white clover sprouts (6D), which showed 115.6% biomass accumulation. Similarly, Mastropasqua et al. (2020) observed an increase in dry mass accumulation in radish, soy, mung beans, and pumpkin sprouts grown for 5 days in darkness conditions [16]. On the 5th and 7th days of harvest, there were no visible differences between the control and darkness samples in the biomass of the white clover sprouts.

The germination rate of the tested clover sprouts after 72 h was 77% for control (L), 85% for darkness conditions (D), 86% for standard light and microgravity (LM), and 87% for darkness and microgravity (DM). These results suggest that microgravity and darkness conditions during the first 3 days of the white clover seed germination caused their increased germination rate compared to control conditions, i.e., standard light and normal gravity. Nakajima et al. (2021) also observed an increased growth of mung bean sprouts in clinostat conditions and suggested that disturbed gravity conditions may enhance the activity of the amylase enzyme, which is responsible for the breakdown of starch, an energy source for growing seeds [8]. In another study, a reduction in the germination of rocket seedlings growing in clinorotation conditions on Earth and on the International Space Station was observed [17]. These contradictory results can be explained by the difference in seed species and the fact that, depending on the species examined, the growth rate in conditions of disturbed gravity may differ. Interestingly, the germination rate of the white clover seeds in the first 3 days was increased in darkness (D) and microgravity (DM and LM) conditions compared to control conditions (L). However, in the following days (5–7), the growth of the sprouts in microgravity conditions (LM and DM) was inhibited in comparison to the control sprouts (L), as shown by the results of biomass accumulation, while the growth of the sprouts in darkness and control conditions on the other hand was accelerated (Figure 2).

### 2.2. Amount of Bioactive Compounds

The results of HPLC quantitative phytochemical analysis indicated that the white clover sprouts contain isoflavones and phenolic acids (Table 2).

As far as isoflavone content is concerned, these results can be compared to our previous study, describing the chemopreventive activity of clover sprouts from four different species [18]. Similarly to that study, it was found that white clover contained isoflavones such as ononin and formononetin, but additionally we also found daidzin. Ononin and formononetin concentrations were significantly higher in our previous study [18]. This discrepancy in the results may be caused by the use of different clover varieties; in this study, we used *Trifolium nano repens* seeds, and in the previous study, *Trifolium repens* Grasslands variety was used. Generally, in the simulated microgravity samples (5LM, 7LM, 5DM, 6DM, and 7DM) the sum of isoflavones increased in comparison to the samples grown in normal gravity (5L, 7L, 5D, 6D, and 7D). The amount of isoflavones in white clover sprouts varied from 7.65 to 28.26 mg/100 g dw and the sum of isoflavones was the highest in sample 5LM grown in simulated microgravity and standard light conditions. It is worth mentioning that this amount was almost two times higher than in control sprouts (5L), the same observation was in samples 5D and 5DM. What is important, the same sample (5LM) had the lowest amount of phenolic acids. Interestingly, a significant increase in ononin synthesis was observed in the sprouts from simulated microgravity conditions, despite the light impact. Levine et al. (2001) also found that simulated microgravity can enhance the level of isoflavones in 6-days-old soybean seedlings [19].

As we could not find any results describing the phenolic acid amount in white clover sprouts, we can only compare our results to the results from white clover leaves or flowers. Phenolic acids, such as protocatechuic and caffeic acid, were present in the studied sprouts, with the predominance of the latter, similarly to Kicel et al. (2006). Moreover, we noted a presence of quercetin same as Kicel et al. (2012) and Tundis et al. (2015) and chlorogenic acid, the presence of which in white clover, to our knowledge, was not yet reported [20,21,22]. The sum of phenolic acids in white clover sprouts varied from 33.2 to 268.9 mg/100 g dw. These values significantly exceed the results obtained for white clover leaves by Popovic et al. (2014) [23]. The samples with the highest amount of phenolic compounds like 6D, 7DM, and 5DM were grown in darkness and two of them (5DM and 7DM) additionally in simulated microgravity conditions, which may suggest that the absence of light and disturbed gravity may be stress factors that influence the increased synthesis of the mentioned compounds in white clover sprouts. Similarly, Nakajima et al. (2019) discovered an increased concentration of phenolic compounds in the mung bean seedlings grown in disturbed gravity with the use of clinostat [9].

The samples with the lowest content of the active compounds were 6DM, 5D, and 6D for isoflavones and 5LM and 6LM for phenolic acids, respectively. It can be concluded that total darkness during the sprouting period contributes to a decreased synthesis of isoflavones in white clover sprouts and at the same time, an increased synthesis of phenolic acids. As far as sprouting time is concerned, the sum of phenolic compounds and isoflavones increased along with the days of germination, so the 5-days-old sprouts contain less phenols than the 7-days-old sprouts. The only exception is sample 5LM in which the highest amount of isoflavones was observed on the 5th day of sprouting and on the 6th and 7th days the content decreased.

### 2.3. Abscisic Acid Amount and Phenylalanine Ammonia-Lyase Activity

**Figure 3:**
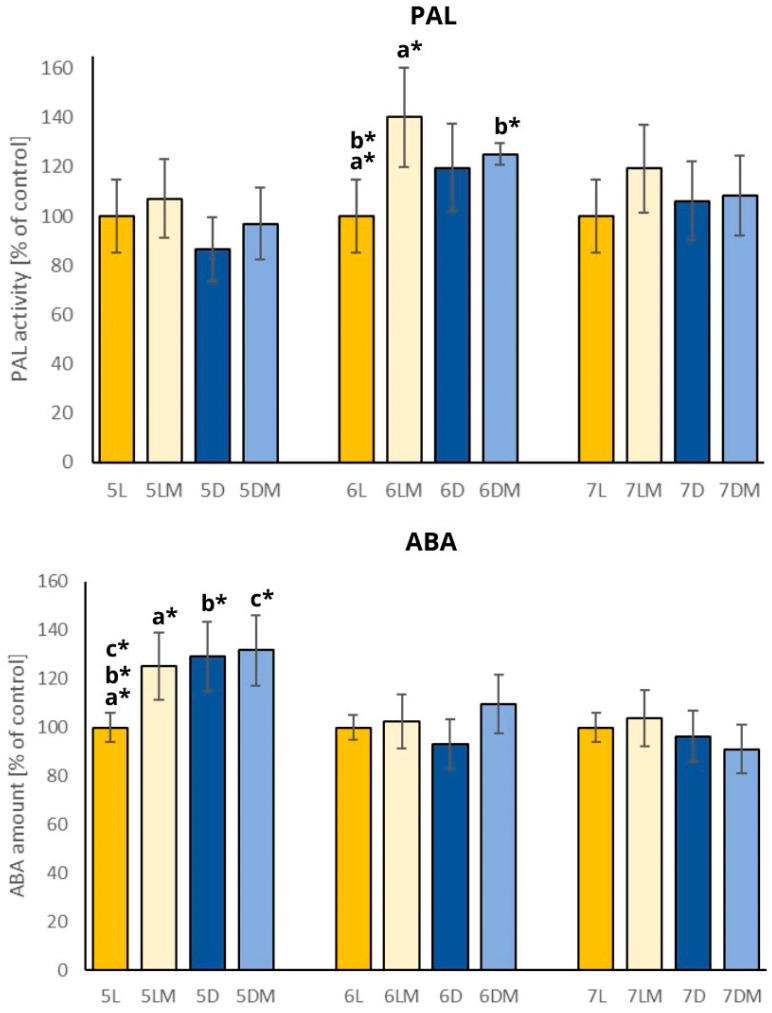
Abscisic acid (ABA) amount and phenylalanine ammonia-lyase (PAL) activity in the white clover sprouts presented as the % of control (control: the amount of ABA and PAL activity in the 5L, 6L, and 7L sprouts). Significant differences were calculated for each day of harvesting separately and were marked with pairs of letters a–c (*: *p* ≤ 0.05). Abbreviations of the sprouts’ samples: L—the sprouts grown in standard light conditions; D—the sprouts grown in darkness; LM—the sprouts grown in standard light and simulated microgravity conditions; DM—the sprouts grown in darkness and simulated microgravity conditions. The numbers placed before each abbreviation indicate harvest days (5, 6, and 7 days).

The results from abscisic acid (ABA) amount and phenylalanine ammonia-lyase (PAL) activity in the examined sprouts are presented in Figure 3.

PAL is the key enzyme involved in isoflavone synthesis in plants [13] and generally, it plays a vital role in phenylpropanoid biosynthesis [24], so it is also responsible for phenolic acid production. PAL activity was measured in this study to assess whether the results of quantitative analysis of isoflavones and phenolic acids coincide with PAL activity and whether this could be a possible explanation for the increased amount of certain compounds in the sprouts. PAL activity increased by about 40% in the 6-days-old white clover sprouts grown in standard light and simulated microgravity conditions (6LM) and about 20% in the 6-days-old sprouts grown in simulated microgravity and darkness conditions (6DM). The 5- and 7-days-old sprouts did not show significant differences in PAL activity between the tested and control sprouts. Our results indicate that the activity of PAL increased, especially in the sprouts cultivated in simulated microgravity conditions and both standard light and darkness conditions. The most significant effect was observed for the 6-days-old sprouts (6LM and 6DM). These results are consistent with those of Wakabayashi et al. (2005), who revealed that disturbed gravity in the form of hypergravity caused significantly higher activity of PAL in wheat seedlings [11]. What is interesting, the increased activity of PAL did not enhance the synthesis of isoflavones and phenolic acids. It is worth mentioning that the sum of isoflavones in the samples with the highest PAL activity (e.g., 6LM or 6DM) was lower than in the control 6L sprouts. Similar results were achieved by Cowles et al. (1984), where the seedlings of *Pinus* sp. grown in microgravity exert increased secondary compound accumulation with decreased PAL activity [25], which may suggest that PAL activity in the case of microgravity-grown plants is not necessarily linked with the accumulation of bioactive compounds.

ABA is a stress-related hormone in plants and its activity is regulated by the environment in response to stress [26]. A significant increase in the ABA amount was observed in the 5-days-old white clover sprouts grown in simulated microgravity conditions both in standard light and darkness, and also the sprouts grown under normal gravity but in darkness conditions. The 6- and 7-days-old sprouts did not show significant differences in ABA amount between the tested and control sprouts. Our results indicate that simulated microgravity and darkness conditions during the sprouting of the white clover sprouts resulted in the increase in ABA level, but statistically significant differences were only observed for the 5-days-old sprouts. This may be due to the fact that the plant has a higher stress level in the initial days of growth (day 5), and in the following days (6 and 7), this level decreases as the plant adapts to the stress conditions. It would be interesting to examine the ABA level also in the 3- or 4-days-old sprouts to compare the effect and confirm this thesis. Our results contradict the results obtained by Schulze et al. (1992), who proved that the content of ABA in 5-days-old *Zea mays* seedlings grown in a space station (microgravity) did not differ significantly as compared to control earth-grown seedlings [27].

### 2.4. Antioxidant Activity

Because of the proven antioxidant activity of isoflavones [28], other phenolic compounds [29], and also the sprouts from the *Fabaceae* family [18,30,31], in the next step of the analysis, we wanted to evaluate the antioxidant potential of the white clover sprouts to check whether the microgravity or darkness can influence this activity.

The results from the FRAP and DPPH assays are presented in Table 3. The most pronounced antioxidant effect was noted for the 5LM, 5DM, 6D, and 7DM sprouts in both tests. This observation can be explained by the concentration of isoflavones and phenolic acids, as these sprouts contained the highest amount of the compounds, e.g., the 5LM sprouts had the highest amount of isoflavones, while the 5DM, 6D, and 7DM sprouts were the richest in phenolic acids. The comparison of our results with those obtained by Galanty et al. (2022) for white clover sprouts showed higher antioxidant activity for control sprouts as well as for the sprouts grown under stress conditions (simulated microgravity and total darkness) [18]. Based on these results, it can be concluded that stress conditions like simulated microgravity and the lack of sunlight during the early stage of sprouting can stimulate the antioxidant activity of the sprouts. These findings are consistent with the results by Nakajima et al. (2019), who studied 4-days-old mung bean seedlings under disturbed gravity conditions with the use of a clinostat [9].

### 2.5. Cytotoxic Activity

**Figure 4:**
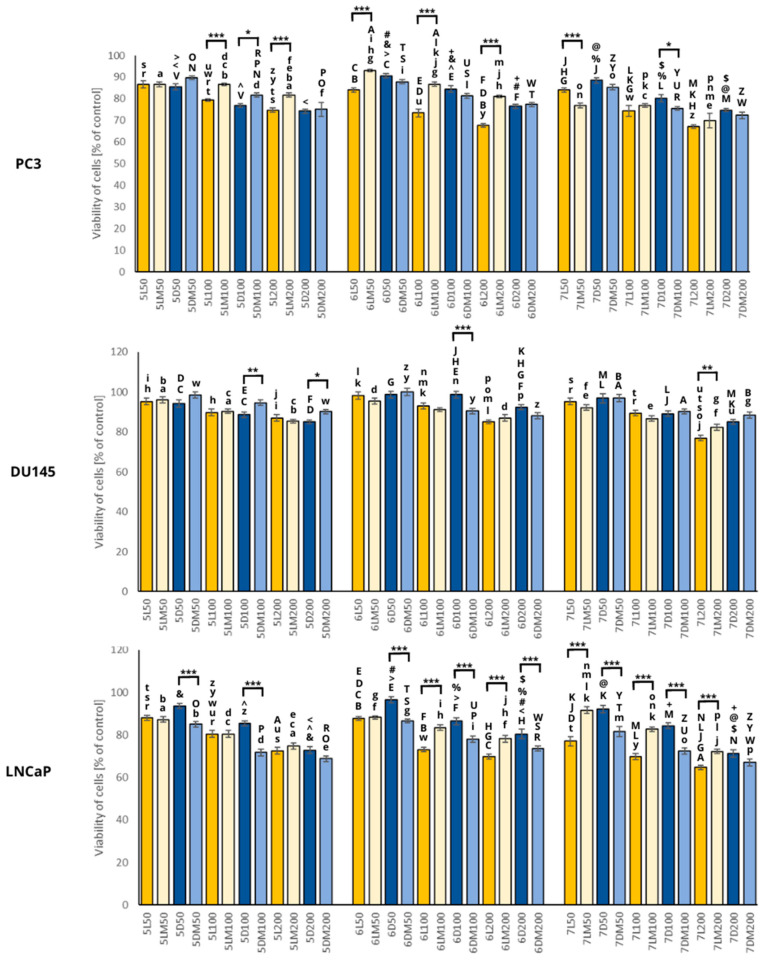
Viability of the PC3, DU145, and LNCaP cells after 24 h incubation with the white clover sprout extracts used in different concentrations (50, 100, and 200 μg/mL). Significant differences between the cells treated with the extracts from the white clover sprouts grown in microgravity conditions versus control sprouts were marked with an upper black line (***: *p* ≤ 0.001; **: *p* ≤ 0.01; *: *p* ≤ 0.05). Significant differences between different days of harvesting (e.g., 5LM50 vs. 6LM50), concentrations (e.g., 5LM50 vs. 5LM100) of extracts, and light conditions (e.g., 5LM50 vs. 5DM50) were marked with pairs of letters a–z, A–Z, and special signs (@, <, >, ^, %, &, #, +, and $). Abbreviations of the sprouts’ samples: L—the sprouts grown in standard light conditions; D—the sprouts grown in darkness; LM—the sprouts grown in standard light and simulated microgravity conditions; DM—the sprouts grown in darkness and simulated microgravity conditions. The numbers placed before each abbreviation indicate harvest days (5, 6, and 7 days). The numbers placed after each abbreviation indicate concentration (50, 100, and 200 μg/mL).

**Figure 5:**
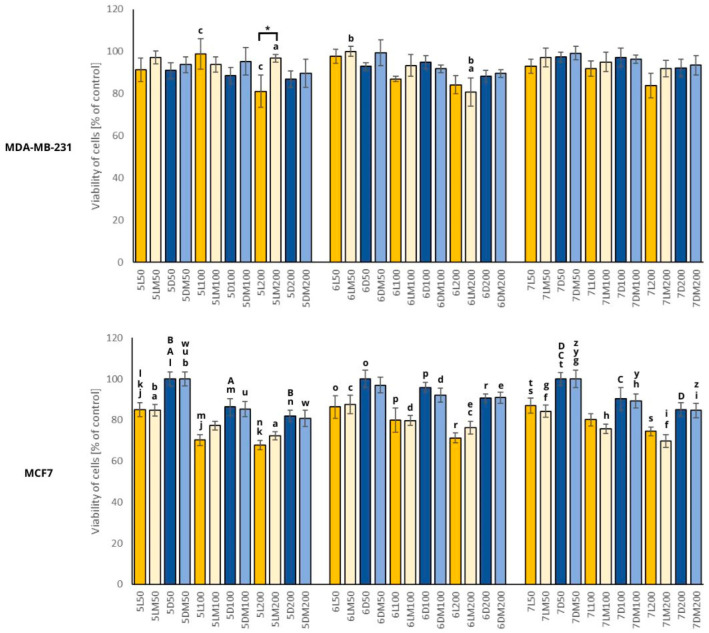
Viability of the MDA-MB-231 and MCF7 cells after 24 h incubation with the white clover sprout extracts used in different concentrations (50, 100, and 200 μg/mL). Significant differences between the cells treated with the extracts from the white clover sprouts grown in microgravity conditions versus control sprouts were marked with an upper black line (*: *p* ≤ 0.05). Significant differences between different days of harvesting (e.g., 5LM50 vs. 6LM50), concentrations (e.g., 5LM50 vs. 5LM100) of extracts, and light conditions (e.g., 5LM50 vs. 5DM50) were marked with pairs of letters a–z and A–D. Abbreviations of the sprouts’ samples: L—the sprouts grown in standard light conditions; D—the sprouts grown in darkness; LM—the sprouts grown in standard light and simulated microgravity conditions; DM—the sprouts grown in darkness and simulated microgravity conditions. The numbers placed before each abbreviation indicate harvest days (5, 6, and 7 days). The numbers placed after each abbreviation indicate concentration (50, 100, and 200 μg/mL).

In order to study the cytotoxic potential of the examined white clover sprouts, we decided to use hormone-dependent cancer cells like breast and prostate cancer. This decision was based on the content of isoflavones, which may play a role in breast cancer prevention [32] and inhibit estrogen-dependent tumorigenesis [33]. Cytotoxic activity of white clover sprouts was reported only in our previous article [18], using the same prostate and breast cancer cell lines. The only differences are the variety of white clover seeds and concentrations of extracts used in the cytotoxicity test. In this study, we used *Trifolium nano repens* and in the previous study, *Trifolium repens* L., Grasslands variety. In Figure 4 and Figure 5, we present the results of the extracts in the concentrations of 50, 100, and 200 μg/mL to visualize the impact of dosage on cytotoxic activity, and only the highest concentrations (100 and 200 μg/mL) exerted an impact on cancer cells. The results obtained in this study showed that white clover sprouts have rather weak activity against breast and prostate cancer as the viability of the cells did not decrease below 60%.

#### 2.5.1. Prostate Cancer

In the case of white clover sprout extracts and their influence on the viability of human prostate cancer cells like PC3, DU145, and LNCaP, the results are presented in Figure 4. Our observations that the androgen-dependent LNCaP cells were the most sensitive to the white clover extracts, and the low metastatic androgen-independent DU145 cells were the most resistant, are consistent with the results obtained in our previous study [18], and the observed effects were dose-dependent.

The most pronounced cytotoxic activity in all cancer cell lines was in sample 7L in the highest concentration (200 μg/mL), which caused a 23–35% reduction in cell viability. The day of harvest did not significantly affect the cytotoxic activity of the sprouts in most cases. As it comes to PC3 cells, the most statistically significant differences between the test and control groups were found in the sprouts grown in standard light, where the control sprouts were more potent in cytotoxic activity in comparison to the sprouts grown in simulated microgravity conditions; the only exception was sample 7LM and 7L at the concentration of 50 μg/mL. It can be observed that the changes in the growth conditions of the sprouts significantly affected their cytotoxic activity against the LNCaP cells. The white clover sprouts grown in simulated microgravity conditions in standard light had reduced cytotoxic activity in comparison to the control sprouts, and the same situation was for the PC3 cells. However, when lighting conditions were changed to darkness, the situation was reversed; the sprouts grown in simulated microgravity conditions showed increased cytotoxic activity against the LNCaP cells compared to the sprouts grown in normal gravity, and the differences were statistically significant.

It can be concluded that simulated microgravity combined with the lack of sunlight during the growth of the white clover sprouts significantly increases cytotoxic activity against the LNCaP cells. The time of sprouting in most cases does not affect cytotoxic activity, but this effect was dose-dependent.

#### 2.5.2. Breast Cancer

The cytotoxic activity of the white clover sprout extracts tested on the breast cancer cell lines MCF7 and MDA-MB-231 are presented in Figure 5. The most active was control sample 5L in the highest concentration (200 μg/mL), which caused a 33% and 19% decrease in the viability of the MCF7 and MDA-MB-231 cells, respectively. There were no significant differences between the cytotoxic activity of the sprouts grown in simulated microgravity conditions and in normal gravity conditions, except the 5LM sample (at the concentration of 200 μg/mL), which was less active compared to control sample 5L toward the MDA-MB-231 cells.

In the case of MCF7 cells, an interesting observation concerning lighting conditions was noted. The sprouts grown in standard light conditions (L and LM) exert more potent cytotoxic activity compared to the sprouts grown in darkness (D and DM), but these differences were not statistically significant. Additionally, the content of isoflavones may have influenced the sprouts’ activity against the MCF7 cells. For example, the samples grown in standard light 5L and 5LM contained 11.6 and 28.26 mg/100 g dw of isoflavones, respectively, and the sprouts grown in darkness 5D and 5DM contained 7.65 and 15.33 mg/100 g dw, respectively, a similar situation is for 6-days-old sprouts. In turn, in the 7-days-old sprouts, there are no visible differences between isoflavone content in standard light and darkness conditions.

The estrogen-independent and highly invasive MDA-MB-231 cells were generally more resistant than the estrogen receptor-positive MCF7 cells, similar to the observation from our previous study [18]. There were practically no differences between control and simulated microgravity-grown sprouts, which means that the latter condition does not influence the cytotoxic properties of white clover sprouts against breast cancer cells.

### 2.6. Safety Studies

**Figure 6:**
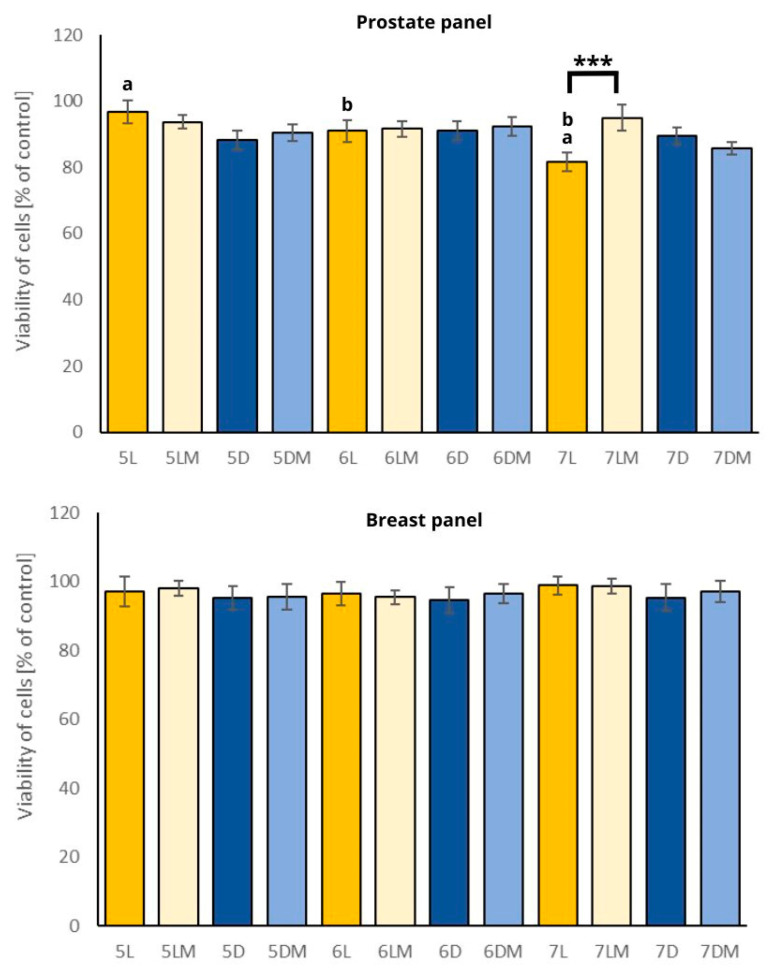
Viability of the PNT2 and MCF10A cells after 24 h incubation with the white clover sprout extracts (200 μg/mL). Significant differences in the viability of the cells treated with the extracts from the white clover sprouts grown in microgravity conditions versus the sprouts grown in normal gravity were marked with an upper black line (***: *p* ≤ 0.001). Significant differences between different days of harvesting (e.g., 5L vs. 6L) were marked with pairs of letters a,b. Abbreviations of the sprouts’ samples: L—the sprouts grown in standard light conditions; D—the sprouts grown in darkness; LM—the sprouts grown in standard light and simulated microgravity conditions; DM—the sprouts grown in darkness and simulated microgravity conditions. The numbers placed before each abbreviation indicate harvest days (5, 6, and 7 days).

As a last step of the experiment, the selectivity and safety studies of the examined sprouts were performed. In Figure 6, the influence of white clover sprout extracts used in the highest concentration of 200 μg/mL on normal human prostate PNT2 and breast MCF10A cell viability is presented. Generally, all the extracts were safe for the PNT2 and MCF10A cells. There were no visible differences between the sprouts grown in simulated microgravity conditions and the sprouts grown in normal gravity, except the 7-days-old sprouts grown in standard light conditions where, the control sprouts (7L) caused a significant decrease in the cell viability of the PNT2 cells in comparison to the sprouts grown in simulated microgravity conditions (7LM). This may suggest that the sprouts selectively, although not strongly, decrease the viability of prostate and breast cancer cells, while the corresponding non-cancerous cells remain unaffected.

## 3. Materials and Methods

### 3.1. Plant Material

In the preliminary research, we tried to cultivate white (*Trifolium nano repens*), Persian (*Trifolium resupinatum*), red (*Trifolium pratense*), and crimson clover (*Trifolium incarnatum*); vetch (*Vicia sativa*); alfalfa (*Medicago sativa*); soy (*Glycine max*); lentil (*Lens culinaris Medik*.); fenugreek (*Trigonella foenum-graecum*); lupine (*Lupinus* L.); green peas (*Pisum sativum*); chickpea (*Cicer arietinum*); mung (*Vigna radiata*); and adzuki beans (*Vigna angularis*). Finally, we decided to use white clover sprouts. The clover (*Trifolium nano repens*) seeds were obtained from TORAF sp. z o.o. company, Kujakowice Górne, Poland (voucher specimen No: C/1 NL3114201) and was deposited in the Department of Food Chemistry and Nutrition. The seeds were soaked for 30 min in mineral water with a known and constant content of 213.4 mg/mL of mineral compounds (bicarbonates 121.1 mg/mL; fluorides < 0.1 mg/mL; magnesium 5.3 mg/mL; calcium 36.3 mg/mL; natrium 7.8 mg/mL). The seeds were transferred to special plastic containers with a capacity of 150 mL, which were previously washed with boiling water to avoid the presence of possible pathogens or fungi and sprayed with a few milliliters of mineral water. After that, the containers with the seeds were placed in the random positioning machine (RPM) in which the containers were rotated throughout the entire cultivation period to simulate the microgravity conditions. Different light conditions were used; one group of sprouts grew in standard light conditions, which means ~12 h of sunlight and ~12 h of night, and the other in complete darkness. The sprouting seeds were watered with 5 mL mineral water once a day, the cultivation temperature was 22 ± 1 °C, and after 5, 6, or 7 days of cultivation period, they were removed from containers, weighed, and frozen in −20 °C. The control sprouts were grown in Easy Green automatic sprouters under the same light and temperature conditions as the experimental sprouts.

Abbreviations were used in this work to denote the sprouts’ samples: L—the sprouts grown in standard light conditions; D—the sprouts grown in darkness; LM—the sprouts grown in standard light and simulated microgravity conditions; DM—the sprouts grown in darkness and simulated microgravity conditions. The numbers placed before each abbreviation indicate harvest days (5, 6, and 7 days).

### 3.2. Microgravity Simulation Setup

The RPM (AATC, [34]) is a small random positioning machine 30 cm × 30 cm × 28 cm presented in a graphical abstract. The device was used to simulate altered gravity conditions according to the method described by Borst and Loon, (2009) [35]. Unlike 3D clinostats, the RPM device more accurately simulates the behavior of biological specimens in free fall conditions detected in space because of the randomization of the rotation movement of two axes. The simulation of microgravity was based on the random deviation of inner and outer ring movement, where the speed of each ring was averaged to 60 rpm. The machine operated using two stepper motors. The frames were mechanically balanced to reduce vibrations. The testing platform allowed for the adjustment of a 400 g payload in the center of the inner ring.

### 3.3. Extracts Preparation

#### 3.3.1. Qualitative and Quantitative Analysis and Antioxidant Activity

The sprouts were defrosted and crushed in a mortar to destroy cell membranes and release active compounds into the extract. Then, the sprout mass was weighed and extracted for 3 h in a Soxhlet apparatus using methanol. A total of 15 mL of each extract was collected and frozen at −20 °C for further analysis. The extracts prepared in this way were used for HPLC analysis and the evaluation of antioxidant capacity (DPPH and FRAP methods).

#### 3.3.2. Cytotoxic Activity

The residues of the above-mentioned extracts were evaporated and dried to dry mass, and then 10 mg of the dry extracts were weighed and dissolved in 1 mL of DMSO. The extracts prepared in this way were used for cytotoxicity tests.

#### 3.3.3. Abscisic Acid Amount and Phenylalanine Ammonia-Lyase Activity

The sprouts were frozen at −80 °C for a week before analysis to destroy plant cell membranes. After that, the sprouts were defrosted and dried with a paper towel to remove any remaining water. In total, 1 g of the sprouts was weighed, and 8 mL of phosphate-buffered saline was added (ratio 1:9) and homogenized (homogenizer CAT X-360, Ingenieurburo CAT, M. Zipperer, Ballrechten-Dottingen, Germany) on ice for 1 min. Then, the homogenates were centrifuged (centrifuge MPW-350R, MPW Med. Instruments, Warsaw, Poland) at 8000× *g* RPM and 4 °C for 20 min, and the supernatants were collected in Eppendorf tubes and frozen at −20 °C for further analysis. Before performing the tests, the extracts were defrosted and centrifuged again at 10,000× *g* RPM and 4 °C for 20 min. The extracts prepared in this way were used for ELISA tests.

### 3.4. Biomass and Germination Rate

Before each cultivation process, the seed mass was carefully weighed. The sprouts were harvested after 5, 6, and 7 days of cultivation and their biomass was weighed. Then, the obtained biomass of the sprouts was calculated as a % of control samples (5L, 6L, and 7L) per 100 g of seeds.

### 3.5. Germination Rate

For the germination rate, 100 white clover seeds were placed in a Petri dish containing gauze moistened with 10 mL of mineral water. The Petri dishes covered with a glass funnel to retain moisture were placed in the same conditions of standard light and darkness (L and D) as mentioned in Section 3.1 for 72 h. In turn, for simulated microgravity conditions, the seeds were placed in the same special plastic containers as those mentioned in Section 3.1 and placed in the RPM setup mentioned in Section 3.2. The seeds were watered every 24 h with a few mL of mineral water. After 72 h, the germinated seeds were counted and the percentage of the germinated seeds was calculated.

### 3.6. Qualitative and Quantitative HPLC Analysis

The quantitative analysis of isoflavones and phenolic compounds in the white clover sprouts was performed on the Dionex HPLC system equipped with a PDA 100 UV-VIS detector and a Hypersil Gold (C-18) column (5 μm, 250 × 4.6 mm, Thermo EC, Waltham, MA, USA), as previously described [36]. All the analyses were performed in three repetitions, and the values were expressed in mg/100 g of dw of the sprouts. The compounds were identified by comparing their retention times with the standards’ retention times. The compound content was calculated by measuring the peak area with respect to the appropriate standard curve.

### 3.7. Antioxidant Activity

The analysis was performed with the use of the DPPH and FRAP methods. DPPH methanolic solution (3.9 mL, 25 mg/L) was mixed with the white clover sprout extracts. The reaction was monitored at 515 nm until the absorbance was constant. All the analyses were performed in three repetitions and the mean capacity was expressed as μM Trolox/100 g dw. FRAP solution (900 μL; 2.5 mL 10 mM ferric-tripiridyltriazine in 40 mM HCl, 2.5 mL 20 mM FeCl_3_·H_2_O, and 25 mL 0.3 mol/L acetate buffer, pH 3.6) was mixed with 90 μL of distilled water and 30 μL of the white clover sprout extracts and measured at 593 nm. All the analyses were performed in three repetitions and the mean results were expressed as μM Fe^2+^/100 g dw. The absorbance of both antioxidant assays was measured using a Biotek Synergy microplate reader (BioTek Instruments Inc., Winooski, VT, USA) [36].

### 3.8. Cell Cultures and Viability Assays

Cytotoxic activity was tested on two panels of human prostate and breast cancer cell lines, including non-cancerous cells for safety studies. Prostate panel: DU-145—androgen-insensitive prostate carcinoma, derived from the metastatic site: brain, ATCC HTB-8; PC3—androgen-insensitive, grade IV prostate carcinoma, derived from the metastatic site: bone, ATCC CRL-1435; LNCaP—androgen-sensitive prostate adenocarcinoma, derived from the metastatic site: lymph node, ATCC CRL-1740; PNT2—prostate epithelial cells, ECACC 95012613. Breast panel: MCF7—ER-positive breast adenocarcinoma, ATCC HTB-22; MDA-MB-231—ER-negative breast adenocarcinoma, ATCC HTB-26; MCF10A—breast epithelial, ATCC CRL-10317. All cell lines were purchased in Merck (Darmstadt, Germany). The cells were grown under standard conditions, as mentioned previously [18]. The cells were seeded in 96-well plates for 24 h (1.5 × 10^4^ cells/well). The culture medium was replaced with a fresh medium containing different concentrations of the extracts tested (10–200 μg/mL, in DMSO) and incubated for 24 h. After that time, the viability of the cells was examined by an MTT assay according to the manufacturer’s instructions. Each experiment was carried out in triplicate. The absorbance was measured at 570 nm using a Biotek Synergy microplate reader (BioTek Instruments Inc., Winooski, VT, USA). Cell viability was expressed as the number of alive cells as a percent of control, untreated cells. Doxorubicin was used as a reference drug.

### 3.9. Abscisic Acid (ABA) Amount and Phenylalanine Ammonia-Lyase (PAL) Activity

ABA amount and PAL activity were measured using ELISA kits (Bioassay Technology Laboratory, Shanghai, China) according to the manufacturer’s instructions. The sprouts’ extracts prepared before (see Section 3.3.3) were used for the analysis, which was performed in three replicates, using a microplate reader (BioTek Instruments Inc., Winooski, VT, USA) and shown as % of control for each day of harvesting (5L, 6L and 7L).

### 3.10. Statistical Analysis

Statistical analysis was performed with Statistica v.13 (Statsoft, Tulsa, OK, USA) tools, using ANOVA one-way analysis of variance and a post hoc Tukey’s test. The use of Student’s *t*-test for a single sample was also used. All the experiments were carried out in triplicate, and the data were reported as the mean ± standard deviation (SD). The differences between the groups were considered statistically significant when the *p*-values were ≤0.05.

## 4. Conclusions

The lack of sunlight and simulated microgravity during growth can be stress factors that affect the biological activity of white clover sprouts. The presented results indicate that simulated microgravity and/or total darkness during the first few days of growth affects various parameters of white clover sprouts, starting from biomass reduction, the increased synthesis of bioactive compounds, and change in the activity of PAL and ABA level, ending with the increased antioxidant and cytotoxic activity in the case of LNCaP cells. The presented results are promising, however, taking into account the fact that, to our knowledge, this study is the first to describe so broadly the impact of microgravity on various aspects: the appearance, biomass, content of active compounds, and biological activity of white clover sprouts, and further research are necessary to draw more clear conclusions and perspectives. Additionally, in relation to our preliminary studies and attempts to cultivate other representatives of the *Fabaceae* family, it can be concluded that this family is difficult to grow in simulated microgravity and has difficulties in the tolerance of such extreme conditions. Therefore, it would be interesting and important to search for this kind of functional food candidate also in other plant families.

## Figures and Tables

**Table 1:** Growth parameters of white clover sprouts harvested for 5, 6, and 7 days in standard light and darkness, in disturbed gravity and control conditions.

Sample	Seedling Length [mm]	Stem Length [mm]	Roots Length [mm]
5L	12.67 ± 2.08 ^cd^	8.67 ± 1.15 ^c^	3.00 ± 2.65
5LM	9.33 ± 0.58	5.33 ± 0.58	2.67 ± 0.58
6L	25.33 ± 0.58 ^ace^	14.33 ± 3.05 ^a^	8.67 ± 2.52
6LM	8.00 ± 1.73 ^a^	4.00 ± 0.60 ^a^	2.00 ± 1.00
7L	28.33 ± 0.58 ^bdfg^	19.67 ± 2.52 ^bcde^	6.33 ± 3.21
7LM	18.00 ± 5.57 ^b^	9.00 ± 1.00 ^b^	7.33 ± 4.73
5D	18.00 ± 6.24 ^jk^	13.00 ± 6.08 ^h^	4.67 ± 1.53
5DM	9.67 ± 2.08	5.67 ± 1.15	2.67 ± 1.15
6D	30.33 ± 1.53 ^hjl^	22.00 ± 4.36 ^fi^	6.67 ± 3.21
6DM	9.00 ± 1.73 ^eh^	6.00 ± 1.73 ^f^	2.00 ± 1.73
7D	46.33 ± 6.80 ^gikl^	32.33 ± 6.43 ^eghi^	11.67 ± 6.66
7DM	14.33 ± 2.08 ^fi^	9.67 ± 0.58 ^dg^	3.33 ± 2.08

Values are presented as the mean (mm) ± SD (standard deviation) of three independent measurements. Significant differences were calculated for each growth parameter separately (each column separately) and presented as the pairs of letters in superscript next to the values if the *p* ≤ 0.05. Abbreviations of the sprouts’ samples: L—the sprouts grown in standard light conditions; D—the sprouts grown in darkness; LM—the sprouts grown in standard light and simulated microgravity conditions; DM—the sprouts grown in darkness and simulated microgravity conditions. The numbers placed before each abbreviation indicate harvest days (5, 6, and 7 days).

**Table 2:** Concentration of isoflavones and phenolic acids in white clover sprouts harvested for 5, 6, and 7 days in standard light and darkness, in disturbed gravity and control conditions.

Sample	Daidzin	Ononin	Formononetin	Isoflavone Sum	Protocatechuic Acid	Caffeic Acid	Chlorogenic Acid	Phenolic Acid Sum
5L	6.76 ± 0.10 ^cij^	0.97 ± 0.02 ^bhij^	3.87 ± 0.40 ^ghi^	11.6	7.73 ± 0.94 ^bfg^	85.52 ± 5.0 ^cj^	38.01 ± 3.90 ^cij^	131.26
5LM	15.14 ± 0.81 ^abcd^	9.49 ± 1.26 ^abc^	3.63 ± 0.20 ^ab^	28.26	9.69 ± 0.75 ^ab^	21.50 ± 1.05 ^abcd^	2.02 ± 0.30 ^abcd^	33.21
6L	9.16 ± 0.80 ^eikl^	2.75 ± 0.20 ^ehkl^	5.50 ± 0.30 ^dgj^	17.41	10.08 ± 0.80 ^df^	100.32 ± 9.50 ^fkl^	66.88 ± 9.20 ^eik^	177.28
6LM	5.58 ± 0.10 ^aef^	4.78 ± 0.30 ^adef^	3.59 ± 0.70 ^cde^	13.95	5.84 ± 0.23 ^acde^	3.59 ± 0.50 ^efi^	39.65 ± 1.40 ^aef^	49.08
7L	7.66 ± 0.20 ^gk^	<LOD ^gik^	7.66 ± 0.60 ^fhjk^	15.32	8.51 ± 1.50	117.46 ± 2.90 ^gjkm^	81.71 ± 11.10 ^gj^	207.68
7LM	5.30 ± 0.40 ^bgh^	8.29 ± 0.70 ^dg^	5.07 ± 0.40 ^acf^	18.66	8.29 ± 0.97^c^	87.09 ± 6.20 ^begh^	60.83 ± 5.90 ^bgh^	156.21
5D	4.95 ± 0.64 ^jnrs^	<LOD ^jo^	2.70 ± 0.10 ^in^	7.65	5.40 ± 0.45 ^ghk^	84.53 ± 6.30 ^ot^	56.95 ± 6.60 ^op^	147.78
5DM	2.59 ± 0.35 ^dmn^	11.55 ± 0.35 ^cmno^	1.19 ± 0.1 ^blmn^	15.33	10.75 ± 0.60 ^h^	150.32 ± 2.80 ^dno^	55.95 ± 6.0 ^dl^	218.21
6D	2.65 ± 0.10 ^lrt^	<LOD ^lr^	5.30 ± 0.90 ^p^	7.95	10.60 ± 0.99 ^ikl^	149.68 ± 9.40 ^lrtu^	108.62 ± 4.60 ^kno^	268.9
6DM	2.36 ± 0.30 ^fo^	3.37 ± 0.30 ^fmpr^	2.52 ± 0.33 ^elop^	8.25	8.08 ± 0.75 ^ei^	105.32 ± 10.40 ^inpr^	63.97 ± 8.60 ^fmn^	178.38
7D	7.10 ± 0.20 ^pst^	<LOD ^s^	4.74 ± 0.48 ^kr^	11.86	7.12 ± 0.64 ^jl^	79.08 ± 7.20 ^msu^	86.20 ± 6.80 ^p^	172.4
7DM	8.77 ± 0.30 ^hmop^	2.34 ± 0.20 ^nps^	5.26 ± 0.40 ^mor^	16.37	9.64 ± 0.41 ^j^	164.83 ± 9.10 ^hps^	87.97 ± 9.60 ^hlm^	263.32

<LOD—lower than the detection limit. Values are presented as the mean (mg/100 g dw) ± SD (standard deviation) of three independent experiments. Significant differences were calculated for each compound separately (each column separately) and presented as the pairs of letters in superscript next to the values if the *p* ≤ 0.05. Abbreviations of the sprouts’ samples: L—the sprouts grown in standard light conditions; D—the sprouts grown in darkness; LM—the sprouts grown in standard light and simulated microgravity conditions; DM—the sprouts grown in darkness and simulated microgravity conditions. The numbers placed before each abbreviation indicate harvest days (5, 6, and 7 days).

**Table 3:** Antioxidant activity of white clover sprouts harvested for 5, 6, and 7 days in standard light and darkness, in disturbed gravity and control conditions.

Sample	FRAP [mM Fe^2+^/100 g dw]	DPPH [mM TROLOX/100 g dw]
5L	4.34 ± 0.10 ^ad^	1.70 ± 0.15 ^aef^
5LM	6.44 ± 0.20 ^abcp^	2.79 ± 0.10 ^abcd^
6L	5.38 ± 0.10 ^eg^	2.19 ± 0.04 ^gi^
6LM	3.13 ± 0.20 ^befr^	1.58 ± 0.06 ^bgh^
7L	4.75 ± 0.20 ^i^	2.32 ± 0.10 ^el^
7LM	4.35 ± 1.20 ^ch^	2.01 ± 0.21 ^cjk^
5D	3.33 ± 0.17 ^jlp^	1.45 ± 0.12 ^dmo^
5DM	6.21 ± 0.20 ^djk^	2.67 ± 0.11 ^fmn^
6D	6.58 ± 0.10 ^glmnr^	3.14 ± 0.42 ^hiops^
6DM	4.66 ± 0.10 ^fkm^	2.05 ± 0.14 ^npr^
7D	3.43 ± 0.20 ^ino^	1.31 ± 0.12 ^klst^
7DM	5.20 ± 0.10 ^ho^	2.76 ± 0.15 ^jrt^

Values are presented as the mean (mM Fe^2+^/100 g dw or mM TROLOX/100 g dw) ± SD (standard deviation) of three independent experiments. Significant differences were calculated for each assay separately (each column separately) and presented as the pairs of letters (a–t) in superscript next to the values if the *p* ≤ 0.05. Abbreviations of the sprouts’ samples: L—the sprouts grown in standard light conditions; D—the sprouts grown in darkness; LM—the sprouts grown in standard light and simulated microgravity conditions; DM—the sprouts grown in darkness and simulated microgravity conditions. The numbers placed before each abbreviation indicate harvest days (5, 6, and 7 days).

## Data Availability

The data presented in this study are available upon request from the corresponding author.
